# Development of a Specific Mini-Barcode From Plastome and its Application for Qualitative and Quantitative Identification of Processed Herbal Products Using DNA Metabarcoding Technique: A Case Study on *Senna*

**DOI:** 10.3389/fphar.2020.585687

**Published:** 2020-12-17

**Authors:** Xiaolei Yu, Wei Tan, Han Gao, Lin Miao, Xiaoxuan Tian

**Affiliations:** State Key Laboratory of Component-based Chinese Medicine, Tianjin University of Traditional Chinese Medicine, Tianjin, China

**Keywords:** mini-barcode, plastome, metabarcoding, species identification, Herbal products, *Senna*

## Abstract

Herbal products play an important role globally in the pharmaceutical and healthcare industries. However, some specific groups of herbal products are easily adulterated by confused materials on the market, which seriously reduces the products’ quality. Universal conventional DNA barcodes would function poorly since the processed herbal products generally suffer from varying degrees of DNA degradation and DNA mixing during processing or manufacturing. For quality control purposes, an accurate and effective method should be provided for species identification of these herbal products. Here, we provided a strategy of developing the specific mini-barcode using *Senna* as an example, and by coupling with the metabarcoding technique, it realized the qualitative and quantitative identification of processed herbal products. The plastomes of Senna obtusifolia (L.) H.S.Irwin & Barneby and Senna occidentalis (L.) Link were newly assembled, and the hypervariable coding-regions were identified by comparing their genomes. Then, the specific mini-barcodes were developed based on the identified hypervariable regions. Finally, we applied the DNA metabarcoding technique to the developed mini-barcodes. Results showed that the lengths of plastomes of *S. obtusifolia* and *S. occidentalis* were 162,426 and 159,993 bp, respectively. Four hypervariable coding-regions *ycf1*, *rpl23*, *petL*, and *matK* were identified. Two specific mini-barcodes were successfully developed from *matK*, and the mini-barcode of primer 647F-847R was proved to be able to qualitatively and quantitatively identify these two processed *Senna* seeds. Overall, our study established a valuable way to develop the specific mini-barcode, which may provide a new idea for the quality control of processed herbal products.

## Introduction

Herbal products use medicinal plants as raw materials for herbal medicines, herbal extracts and dietary supplements. In recent years, there was a tremendous increase in the global demand of herbal products, making herbal products adulteration and counterfeiting a global problem (Mackey and Liang, 2013; Gromek et al., 2015; Gaudiano et al., 2016). Hence, the authentication of herbal products has become an important topic within and beyond the pharmaceutical and healthcare industries. DNA barcoding is a technique for authenticating species using a standard DNA region, aiming to provide rapid, automatable, and cost-effective methods for accurate identification at the species-level. Studies on DNA barcoding have made remarkable progress in species identification (Naeem et al., 2014; Zhang et al., 2016; Viglietti et al., 2019). Initially used as an identification tool, DNA barcoding is now applied in the industrial quality assurance context to identify herbal products (Mosa et al., 2018; Amritha et al., 2020). However, DNA barcoding faces practical limitations that exclusively restrict it to identifying single ingredient herbal products. Once the plants undergo a series of extraction and processing steps resulting in DNA mixing, DNA barcoding would not be a suitable choice. Unfortunately, herbal products generally suffer from varying degrees of DNA mixing during processing or manufacturing. For a suitable solution to this case, DNA metabarcoding appeared to be an alternative choice.

DNA metabarcoding, combining the next-generation sequencing and DNA barcoding, enables simultaneous multi-taxa identification by using the total DNA extracted from complex samples containing DNA from different origins (Taberlet et al., 2012; Staats et al., 2016). It has generally been assumed that the proportion of reads obtained for a given species is proportional to the contribution of species biomass (Amend et al., 2010; Egge et al., 2013). Hence, species reads obtained from DNA metabarcoding can reflect the richness of species in the community to a certain extent, which makes DNA metabarcoding a widely used tool for biodiversity studies (Egge et al., 2013; Klindworth et al., 2013; Duke and Burton, 2020). Nowadays, DNA metabarcoding has also proved to be applicable for authentication of species diversity in herbal products, and has been used to investigate the level of discrepancy between the expected and detected plant species in the herbal product market (Cheng et al., 2014; Coghlan et al., 2015; Ivanova et al., 2016). However, although DNA metabarcoding is superior to DNA barcoding in the assessment of complex herbal mixtures, it still faces some limitations similar to DNA barcoding. For instance, the selection of barcodes has always been a challenge. Barcodes that are too short may not provide sufficient resolution for identification of multiple-taxa, e.g., P6 loop of *trnL* intron (Taberlet et al., 2007). In contrast, conventional barcodes like *matK*, *rbcL*, *trnH-psbA*, and *ITS2* are generally longer than 500 bp, which is very unfavorable for PCR amplification when facing DNA degraded samples including the processed herbal products (Gao et al., 2019). In this case, a short but informative DNA barcode could be a solution, and that is called a mini-barcode.

The DNA mini-barcode is a short DNA fragment, 100–250 bp in length, with sufficient variable sites for species identification (Little, 2014b). Due to the significantly reduced length of barcode regions, PCR amplification success could be much improved, but the barcode resolution would thus be limited. So, it is necessary to design a specific mini-barcode for accurate species identification of close-related species. For angiosperms, it is now realistic to find a specific DNA mini-barcode by searching the whole plastome owing to the ease of next-generation sequencing (Dong et al., 2013). The plastomes of most land plants exhibit a typical quadripartite structure with stable gene content and gene order (Li et al., 2017). Owing to their characteristics of maternally inherited, multi-copy, and moderate evolutionary rate, the plastome sequences are widely used for molecular marker development (Park et al., 2018; Yu et al., 2019; Li et al., 2020). Moreover, the abundant interspecific sequence diversity makes the plastome sequence a particularly useful tool for providing high-resolution barcodes for close-related species (Jiao et al., 2019; Xia et al., 2019)*.* The utility of mini-barcodes has been successfully demonstrated in a number of specific taxa, such as *Panax* (Dong et al., 2014), *Hypericum* (Costa et al., 2016), and *Phyllanthus* (Srirama et al., 2014)*.*

The seed of Senna obtusifolia, called Juemingzi in China, is regarded as a dual-use material for food and medicine by China Food and Drug Administration. Studies on Juemingzi showed that it has various pharmaceutical properties such as hypertension regulation (Li and Guo, 2002), hepatoprotective effect (Kim et al., 2009) and eyesight improvement (Yang et al., 2012), which have made it a very popular herbal product in China and some other Asian countries. Since the wide applications in pharmaceutical and healthcare industries, the demand for Juemingzi increased rapidly, and the proportion of its adulterant increased at the same time. Among these adulterants, the most common and indistinguishable one is the seed of *S. occidentalis*. However, just like the conditions of other herbal products generally suffering from varying degrees of DNA degradation during processing, the conventional DNA barcodes for Juemingzi was difficult to amplify as well. Moreover, mixing seeds could make DNA barcoding function poorly. Therefore, it is necessary to design the specific mini-barcode and combine with the metabarcoding technique for quality control of Juemingzi.

The aims of our study included: 1) to provide a strategy of developing the specific mini-barcode using *Senna* as an example; 2) to test the feasibility of combining mini-barcodes and DNA metabarcoding techniques for qualitative and quantitative identification of processed herbal products. In this study, the plastomes of *S. obtusifolia* and *S. occidentalis* were first assembled, then the hypervariable coding-regions were further sought out by comparing their genomes. Subsequently, the length and position of suitable mini-barcodes were determined, and finally, we applied DNA metabarcoding techniques to the developed mini-barcodes.

## Materials and Methods

### Plant Material

The fresh plant of *S. obtusifolia* was collected from the Medicinal Botanical Garden of Tianjin University of Traditional Chinese Medicine, Tianjin City (117.06°E, 38.96°N), China. The ungerminated seed of *S. occidentalis* was collected from Baoding City (115.33°E, 38.42°N), China. They were identified by Prof. Tianxiang Li from School of Chinese Materia Medica, Tianjin University of Traditional Chinese Medicine. The voucher species were deposited in Tianjin State Key Laboratory of Modern Chinese Medicine, Tianjin University of Traditional Chinese Medicine, and the voucher numbers were JMZ201806 (*S. obtusifolia*) and WJN201808 (*S. occidentalis*). The Extract Plant DNA kit (Sangon Biotech Co., Ltd., Shanghai, China) was used to extract the total genomic DNA from the fresh leaves of *S. obtusifolia* and the whole seed of *S. occidentalis*. In addition, four batches of processed seeds of *S. obtusifolia* and *S. occidentalis* were purchased from Anguo medicine market to construct experimental mixtures. In particular, the seeds were identified by Prof. Tianxiang Li to avoid the possibility of adulteration. DNA purity was checked using NanoPhotometer®spectrophotometer (IMPLEN, CA, United States). Concentrated DNA was measured using Qubit^®^ DNA Assay Kit in Qubit^®^ 2.0 Fluorometer (Life Technologies, CA, United States). Sequencing library was generated using Truseq Nano DNA HT Sample preparation Kit (Illumina United States) following the manufacturer’s recommendations. The library was sequenced by Illumina HiSeq X Ten platform (Novogene, Nanjing, China) and 150 bp paired-end reads were generated.

### Plastome *de Novo* Assembly and Annotation

Raw data of fastq format of DNA was processed by removing adapter sequences, removing reads with the ratio of N (N indicates that base information cannot be determined) greater than 10%, and removing low-quality reads in which >50% of the bases had a quality value Qphred<=5. Subsequent analyses were based on the filtered high-quality sequences. The plastomes of *S. obtusifolia* and *S. occidentalis* were assembled via combination of *de novo* and reference-guided assembly approaches following the procedure described by Niu *et al* (Niu et al., 2017). The plastome of *S. tora* (NCBI accession number NC_030193) was used as a reference. Then, the finished plastomes of *S. obtusifolia* and *S. occidentalis* were annotated using GeSeq (Tillich et al., 2017), coupled with manual corrections for start and stop codons. Finally, the plastomes of *S. obtusifolia* and *S. occidentalis* were visualized using OGDRAW (Lohse et al., 2013).

### Identification of Hypervariable Coding-Regions and Design of Primers

To find the hypervariable coding-regions, the protein-coding genes of two plastomes were respectively extracted and aligned using PhyloSuite_v1.1.15 (Zhang et al., 2020). Then, the nucleotide variability (Pi) values of protein-coding genes were calculated using DnaSP version 6.11.01 software (Rozas et al., 2017). The regions with high Pi values were selected as the candidate regions for mini-barcode development. The Primer Premier V6.0 was used to design primers for the mini-barcode, and the parameters were as follows: product size between 150 and 300 bp, primer size between 18 and 30 bp, melting temperature (Tm) between 40° and 70°C, GC content between 30 and 70%. Then, the physicochemical properties of the designed primers such as hairpin structure, primer dimer, and annealing temperature were evaluated using Oligo seven software, and primers that were likely to have hairpin structures, primer dimers, or excessive annealing temperature were abandoned.

### Experimental Mixtures Construction, Next-Generation Sequencing, and Analysis of Amplicon Sequence Variants (ASVs)

To test the qualitative and quantitative capacity of developed mini-barcodes in the processed mixture of *S. obtusifolia* seeds and *S. occidentalis* seeds, we prepared four experimental mixtures (for the biomass of each species in the four experimental mixtures, please see Supplementary Table S1). Each experimental mixture contained processed seeds (seeds were fully crushed by a powder mill to facilitate sample mixing) of *S. obtusifolia* and *S. occidentalis.* Then, the genomic DNA was extracted from each experimental mixture using the Extract Plant DNA kit (Sangon Biotech Co., Ltd., Shanghai, China), respectively. The target regions were amplified using two pairs of fusion primers with matching tag sequences (for detailed tag sequences, please see Supplementary Table S2) to ensure that tag jumps would not result in false assignments of sequences to samples (Schnell et al., 2015). Then, PCR reaction was conducted in a 25 μL reaction with 12.5 μL of 2 × Gflex PCR Buffer (containing 1 mM Mg^2+^ and 200 μM dNTP), 0.5 μL of each primer, 0.5 μL Tks Gflex™ (Takara Biomedical Technology Co., Ltd., Beijing, China) DNA Polymerase (1.25 units/μl), 2 μL template DNA, and approximately 9 μL ddH_2_O. The PCR protocol was as follows: preheating at 94°C for 1 min, 30 cycles at 98°C for 10 s, annealing at 55°C for 15 s and elongation at 68°C for 30 s, and final extension at 68°C for 5 min. The negative controls were included in every run. Then, the PCR products were detected on 2% agarose gels. Subsequently, equimolar concentrations of PCR products were pooled to construct the library. Finally, the library was sequenced with 2 × 150 bp reads on the Illumina Hiseq X Ten platform.

The fastq-multx (Aronesty, 2013) was used to split the generated data according to the tag sequences. Then, the primer sequences were trimmed using Cutadapt (Kechin et al., 2017). To construct ASVs (sequences with 100% similarity will be assigned into each ASV), denoize and quality control were performed using DADA2 (Callahan et al., 2016). Then, BlastN was used to search the sequences of ASVs from the chloroplast genomes of *S. obtusifolia* and *S. occidentalis*. Finally, the relationship between species reads and species biomass was analyzed using R package ampvis2 (Andersen et al., 2018).

## Results

### Plastome Features

**FIGURE 1:**
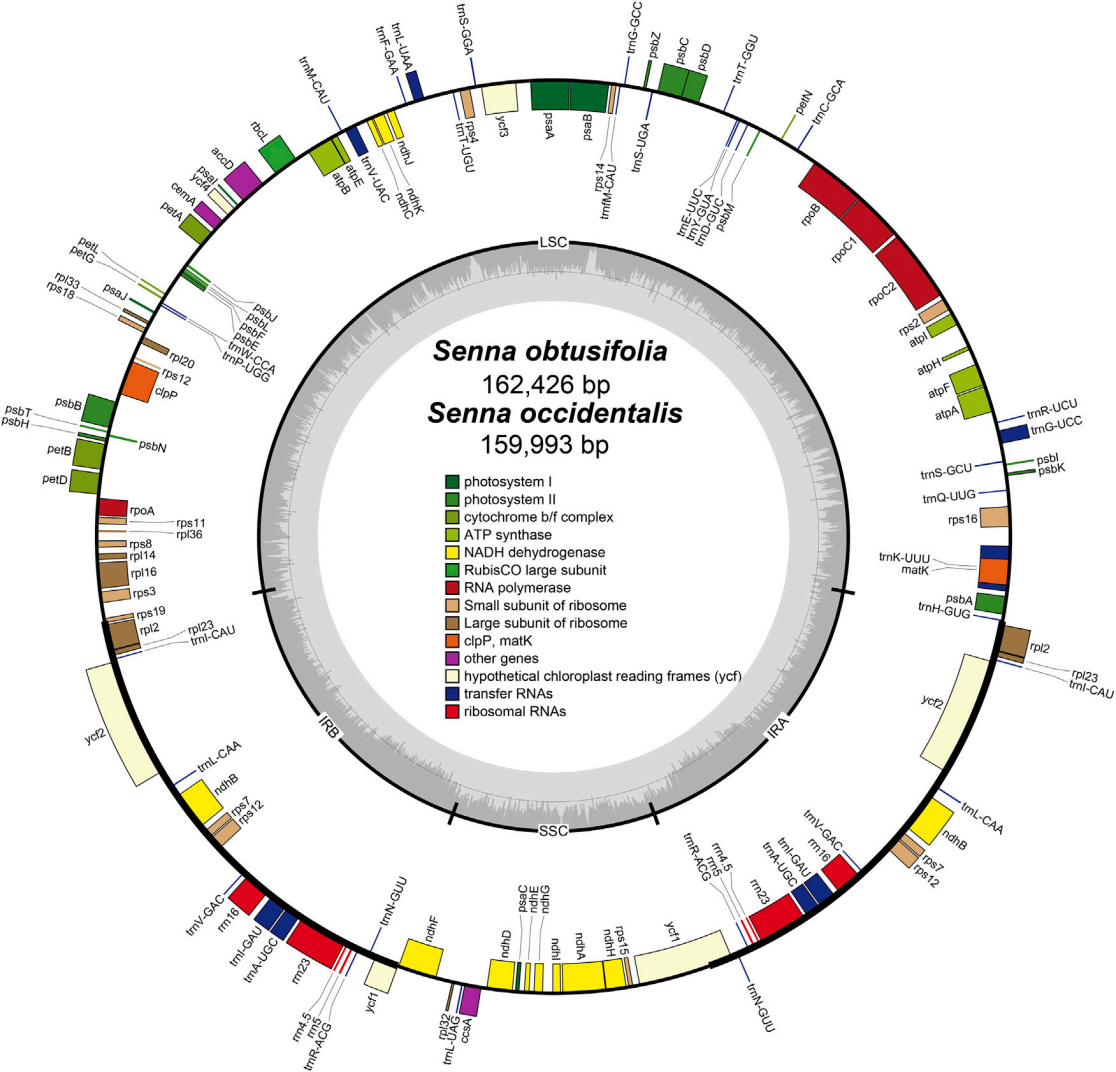
Plastome map of *S. obtusifolia* and *S. occidentalis*. Genes inside the circle are transcribed clockwise, whereas those on the outside are transcribed counter-clockwize. Genes belonging to different functional groups are color-coded. The darker gray in the inner circle represents the GC content, while the lighter gray represents the AT content.

The plastomes of *S. obtusifolia* and *S. occidentalis* were 162,426 and 159,993 bp in length, respectively (Figure 1). Either of the two plastomes displayed a typical quadripartite structure consisting of a pair of IR regions (26,791 bp in *S. obtusifolia*, 26,101 bp in *S. occidentalis*) separated by the LSC (90,843 bp in *S. obtusifolia*, 89,322 bp in *S. occidentalis*) and SSC (26,791 bp in *S. obtusifolia*, 26,101 bp in *S. occidentalis*) regions. Both plastomes exhibited a low GC content (36.0% and 36.2% in *S. obtusifolia* and *S. occidentalis*, respectively). The two *Senna* plastomes encoded an identical set of 129 predicted functional genes, 111 of which were unique, and 18 were duplicated in the IR regions. Overall, the genome structure, gene type, gene content, and GC content of the two *Senna* plastomes were highly similar.

### Selection of Hypervariable Coding-Regions and Design of Primers

**FIGURE 2:**
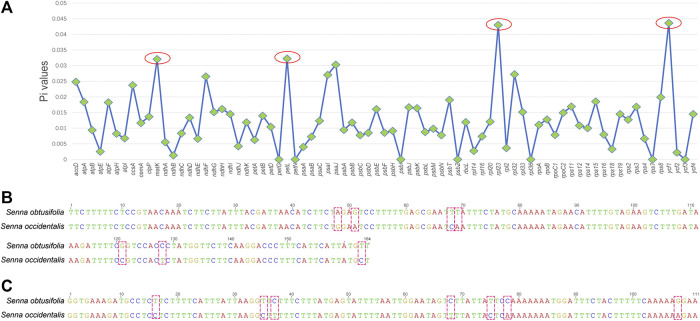
Selection of hypervariable coding-regions and examination of variable sites in two mini-barcodes **(A)** The Pi values of the shared 77 protein-genes of two plastomes **(B)** The mini-barcode of primer 647F-847R, red dotted frames present variable sites **(C)** The mini-barcode of primer 478F-629R, red dotted frames present variable sites.

To find the hypervariable coding-regions for mini-barcode development, the Pi values of the shared 77 protein-genes of two plastomes were calculated using DnaSP. As shown in Figure 2A, *ycf1* had the highest Pi values (0.04363), followed by *rpl23* (0.04301), *petL* (0.03226), and *matK* (0.03206). These observed hypervariable genes were regarded as the candidate regions for mini-barcode development. Then, the primers for the mini-barcode were designed based on these selected hypervariable regions. As depicted in Table 1, two primer pairs were successfully designed from *matK*. However, limited by the length and variability of the barcode regions, as well as the physicochemical characteristics and conservation of the primers, no suitable primers were designed from *rpl23*, *petL* and *ycf1*. The amplicon sizes for primer 647F-847R and 478F-629R were 200 and 151 bp, respectively (Figures 2B,C). Both mini-barcode of primer 647F-847R and 478F-629R had seven variable sites, suggesting a great capacity for identifying the two different *Senna* species.

**TABLE 1:** Primers developed from *matK.*

Primer name	647F-847R	478F-629R
Forward primer sequence 5′ to 3	GTGAATACGAATCTATCT	GTT​CAA​ACC​CTT​CGA​TAC​TG
Reverse primer sequence 5′ to 3	GGATTTTCCTTGATATCT	GGA​ACA​GGA​AAA​ATC​TTG​GA
Amplicon size (bp)	200	151
Variable sites of mini-barcode	7	7
Sequence excluding primers (bp)	164	111
GC% (For/Rev)	37.5/31.2	33.3/33.3
Tm (For/Rev)	45.2/42.4	45.7/45.5

### Qualitative and Quantitative Identification of Two *Senna* Species in Experimental Mixtures by DNA Metabarcoding Technique

Two designed primer pairs were used to amplify the barcode regions in four experimental mixtures. Illumina sequencing results of the amplified products showed that primer 647F-847R generated 1,790,393 reads, which were subsequently clustered into two ASVs (Table 2). These two ASVs were identified as *S. obtusifolia* (ASV1) and *S. occidentalis* (ASV2), with 100% similarity against the corresponding barcode regions. Similarly, for primer 478F-629R, a total of 582,284 reads were generated and clustered into two ASVs, which were identified as two *Senna* species (100% similarity) (Table 2). These two primer pairs successfully amplified the target barcode regions in four experimental mixtures, indicating a qualitative capacity of two mini-barcodes in identifying these two *Senna* species.

**TABLE 2:** BLAST results and sequencing reads of ASV.

Primer name	Sequencing reads of ASV in four experimental mixtures					Blast result	Identity (%)
	ASV	JM1	JM2	JM3	JM4				
647F-847R	ASV1	56,065	564,176	11,901	400,029	*C. occidentalis*	100
	ASV2	57,978	31,078	321,102	348,064	*C. obtusifolia*	100
478F-629R	ASV3	108,018	288,609	6,666	65,827	*C. occidentalis*	100
	ASV4	27,160	2,937	79,349	3,718	*C. obtusifolia*	100

**FIGURE 3:**
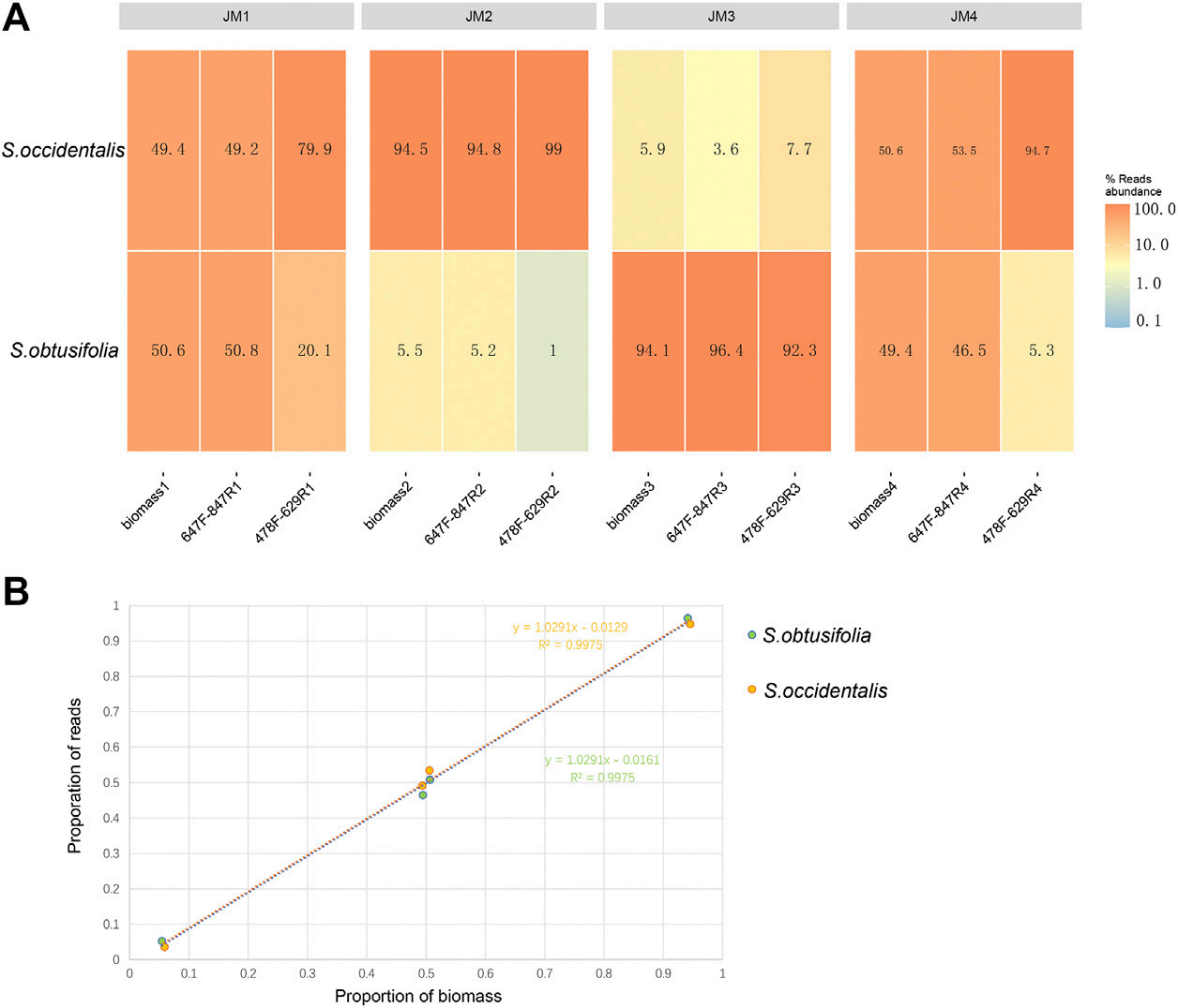
Analysis of species reads abundance and species biomass abundance **(A)** Heat map of reads abundance of two primer pairs in four experimental mixtures **(B)** For primer 657F-858R, the correlation analysis of species reads proportion and species biomass proportion in four experimental mixtures. *X*-axis, proportion of biomass; *Y*-axis, proportion of reads.

To validate the quantitative capacity of the mini-barcodes, the relationship between species reads proportion and species biomass proportion was evaluated in four experimental mixtures (Figure 3A). For primer 478F-629R, there was a significant difference between species reads proportion and species biomass proportion in JM1 and JM4, suggesting a poor quantitative capacity of this mini-barcode in identifying two *Senna* species. For primer 647F-847R, the species reads proportion were almost identical to species biomass proportion in four experimental mixtures. Further correlation analysis (Figure 3B) showed a significant correlation of species reads proportion and species biomass proportion in four experimental mixtures (*R* ^2^ = 0.9975 in two *Senna* species), which indicated that the mini-barcode of primer 647F-847R demonstrated a relatively accurate quantitative ability in identifying two *Senna* species.

## Discussion

Practically, the length of a barcode has always been an issue of concern. A barcode can be generally classified into four types according to its length, for example, micro-barcode within 100 bp (Taberlet et al., 2007), mini-barcode of 100–250 bp (Little, 2014b), standard barcode of 400–800 bp (Hebert et al., 2003), and the whole genome as a super-barcode (Chen et al., 2018). However, micro-barcodes can provide limited resolution for species identification. Although the standard barcode and super-barcode can provide strong resolution, they are difficult to be obtained from the degraded samples due to length limitation. Compared with the three types mentioned above, mini-barcode is the best choice for identifying processed and confused herbal products due to its sufficient variable sites and short in length. However, mini-barcode of high resolution is not easily found, and that is why the whole genome is indispensable for the development of mini-barcodes.

An ideal mini-barcode should meet two requirements: one is a barcode region with sufficient variable sites and short in length, and the other is a primer pair with conservation and meeting physicochemical properties. It is necessary to incorporate the physicochemical properties of the primer into consideration because the hairpin structure, primer dimer, and annealing temperature are all important factors that affect the success of primer amplification (Xie et al., 2019). In addition, insertion and deletion of nucleotides, as well as excessive length variations, are frequently observed in the intergenic region of the chloroplast genome (Bortiri et al., 2008; Liu et al., 2019), which is not conducive to primer conservation. Moreover, the intra-specific variation in the intergenic region is much larger than that in the coding-region (Jiang et al., 2017). Any intra-specific variation may affect the quantitative capacity of the mini-barcode when there are multiple individuals of target species in the mixture. Therefore, the intergenic region was excluded when screening potential DNA barcode regions based on the chloroplast genome.

*mat*K is a recommended gene as the candidate DNA barcode because of its high evolution rate (Lahaye et al., 2008). At the family and genus levels, *matK* provides the high support value of phylogenetic tree and the high-resolution of species discrimination in a given taxon (Ji et al., 2007; Sun et al., 2012). In some groups like *Juglans* (Stanford et al., 2000) and *Fagopyrum* (Ohsako and Ohnishi, 2000), *matK* has also played a useful role in studying intra- and interspecific phylogeny. However, a standard barcode length of *matK* is approximately 800 bp, which is not suitable for identifying severely degraded samples. So, there have been studies focused on the development of mini-barcodes from *matK* and have achieved good results in some specific taxa. For example, Costa et al. (2016) suggested that *matK* was an adequate mini-barcode region for the differentiation of two *Hypericum* species in herbal infusions. Little (2014a) developed a 166 bp mini-barcode from *matK* to authenticate *Ginkgo biloba* herbal dietary supplements. Our study developed two specific mini-barcodes and successfully applied them to identify two *Senna* species, which once again verified the feasibility of developing a specific mini-barcode from *matK*. We recommend that *matK* can be regarded as a preferred option for specific mini-barcode development in subsequent studies.

Primer bias is a well-known factor that substantially influences results in metabarcoding studies. For instance, primer bias has been shown to skew the relative abundance of amplified DNA from experimental mixtures such as stonefly species and some terrestrial arthropods (Elbrecht and Leese, 2015; Piñol et al., 2015). Although our results showed that both primer 647F-847R and 478F-629R provided a positive relationship between species reads abundance and species biomass abundance in the experimental mixtures, which was consistent with other metabarcoding studies (Thomsen et al., 2012; Evans et al., 2016). However, the primer 478F-629R exhibited the large difference between observed reads proportion and given biomass proportion in JM1 and JM4 (i.e., biomass1 vs. 478F-629R1 and biomass4 vs. 478F-629R4). This large variability is most likely resulted from primer bias, and primer binding efficiency and inherent species-specific differences could lead to primer bias in metabarcoding studies (Duke and Burton, 2020). Although the quantitative ability of metabarcoding has always been tested due to primer bias (Elbrecht and Leese, 2015; Bista et al., 2018), our results showed that metabarcoding could provide a relatively accurate quantitative relationship between species read abundance and species abundance when there was no primer bias inference (e.g., primer 647F-847R).

The currently available quality control assessment methods for herbal products are mainly chemical methods, such as thin-layer chromatography, high-performance liquid chromatography, and mass spectrometry (Turova et al., 2018; Mukherjee, 2019). However, the chemical properties of herbal products can be affected by many factors, such as processing methods, storage conditions, and geographic location (Parveen et al., 2016), posing difficulties for proper chemical analyses and objective judgment (de Boer et al., 2015). Moreover, chemical methods may not be able to distinguish closely related species since they generally share chemical compounds (Raclariu et al., 2017), making it challenging to find representative chemical markers for authentication. Compared with chemical methods, DNA mini-barcoding can provide stable quality control assessment for herbal products due to the stability of DNA, and its short barcode region can overcome the difficulties caused by DNA degradation during processing and manufacturing of herbal products. Besides, our study has proved that specific mini-barcodes combined with the metabarcoding technique can realize the qualitative and quantitative identification of closely related species (i.e., *S. obtusifolia* and *S. occidentalis*). From an economic perspective, in our experiment, genomic DNA extraction takes about 1 h, PCR amplification takes about 2 h, and sequencing relies on the Illumina HiSeq X Ten platform, which takes about three days. The cost of each sample is about $100, of which the main cost is library construction and data generation (about $80). However, in the past 20 years, the cost per megabase of DNA sequencing has dropped from about $10,000,000 to about $0.01 (National Human Genome Research Institute, 2020), and it will continue to decline in the future. Moreover, with the introduction of portable sequencers, such as MinION and DNBSEQ E series, the sequencing time has been greatly shortened, and even real-time sequencing can be achieved. Thus, it is conceivable that this molecular technique will become an efficient and economical method for the quality control of herbal products driven by technological development in the near future.

It has always been a concern of the herbal products market whether there is adulteration and how much adulteration since it is not uncommon that the adulterants of expensive or shortage materials are not only similar but also cheaper or easily available. A specific DNA mini-barcode combined with the metabarcoding technique can realize the qualitative and quantitative identification of samples with DNA degradation, which is suitable for processed herbal products like *S. obtusifolia* seeds since they are frequently adulterated with *S. occidentalis* seeds and generally suffer from varying degrees of DNA degradation during harvesting, storage and processing. Nevertheless, not all herbal products require the mini barcodes for quality control assessment. Whether a mini barcode or a regular barcode is needed depends largely on the degree of DNA degradation. Besides, whether specific or universal barcodes are required is also a question to be considered since the quantitative ability of barcodes is greatly interfered by primer bias. Therefore, we recommend that in follow-up studies, different types of herbal products need to be evaluated for the degree of DNA degradation and the suitability of primers to find an optimal quality control strategy.

## Data Availability Statement

The datasets presented in this study can be found in online repositories. The names of the repository/repositories and accession number(s) can be found below: https://www.ncbi.nlm.nih.gov/genbank/, MK817504, https://www.ncbi.nlm.nih.gov/genbank/, MK817505.

## Author Contributions

XT and LM designed the study; XY and WT assembled, annotated and analyzed the plastomes; HG and XY performed the experiment; XY drafted the manuscript; XT and LM revised the manuscript.

## Funding

This work is supported by the grants from Science and Technology Program of Tianjin (No.19ZYPTJC00060), China.

## Conflict of Interest

The authors declare that the research was conducted in the absence of any commercial or financial relationships that could be construed as a potential conflict of interest.
