# Supplementary material for: Development of a Specific Mini-Barcode From Plastome and its Application for Qualitative and Quantitative Identification of Processed Herbal Products Using DNA Metabarcoding Technique: A Case Study on Senna
Source: Front Pharmacol. 2020 Dec 17;11:585687. doi: 10.3389/fphar.2020.585687 (PMC7773718; doi:10.3389/fphar.2020.585687)
Supplement: Supplementary file 1 [file table1.docx]

Supplementary Material

Table S1. Biomass for each species included in the four experimental mixtures.

| Experimental mixtures | | Species | |
| --- | --- | --- | --- |
|  |  | *S. obtusifolia* (mg) | *S. occidentalis* (mg) |
| JM 1 | 15.8 | 15.4 |  |
| JM 2 | 1.3 | 22.5 |  |
| JM 3 | 22.4 | 1.4 |  |
| JM 4 | 22.1 | 22.6 |  |

Values are presented in milligrams (mg).

Table S2. Tag sequences of two primers in the four experimental mixtures.

| Primer name | Tag sequences | | | |
| --- | --- | --- | --- | --- |
|  | JM1 | JM2 | JM3 | JM4 |
| 647F-847R | For (5'-3') TGCTCACT | For (5'-3') ATACGCTC | For (5'-3') ATAGAGCG | For (5'-3') ATCACTGC |
|  | Rev (5'-3') TGCTCACT | Rev (5'-3') ATACGCTC | Rev (5'-3') ATAGAGCG | Rev (5'-3') ATCACTGC |
| 56F-206R | For (5'-3') TATACGCG | For (5'-3') TATCTCGC | For (5'-3') TACTGCTG | For (5'-3') TACGAGAC |
|  | Rev (5'-3') TATACGCG | Rev (5'-3') TATCTCGC | Rev (5'-3') TACTGCTG | Rev (5'-3') TACGAGAC |
